# Next-generation anti-CD20 monoclonal antibodies in autoimmune disease treatment

**DOI:** 10.1007/s13317-017-0100-y

**Published:** 2017-11-16

**Authors:** Fanny Huynh Du, Elizabeth A. Mills, Yang Mao-Draayer

**Affiliations:** 1 University of Michigan Medical School, Ann Arbor, USA; 2 Molecular and Behavioral Neuroscience Institute, University of Michigan Medical School, Ann Arbor, USA; 3 Graduate Program in Immunology, Program in Biomedical Sciences, University of Michigan Medical School, Ann Arbor, USA; 4 Department of Neurology, University of Michigan Medical School, 4015 A Alfred Taubman Biomedical Sciences Research Building, 109 Zina Pitcher Place, Ann Arbor, MI 48109-2200 USA

**Keywords:** Anti-CD20, B cell, Multiple sclerosis, Rheumatoid arthritis, Systematic lupus erythematosus

## Abstract

The clinical success of anti-CD20 monoclonal antibody (mAb)-mediated B cell depletion therapy has contributed to the understanding of B cells as major players in several autoimmune diseases. The first therapeutic anti-CD20 mAb, rituximab, is a murine–human chimera to which many patients develop antibodies and/or experience infusion-related reactions. A second generation of anti-CD20 mAbs has been designed to be more effective, better tolerated, and of lower immunogenicity. These include the humanized versions: ocrelizumab, obinutuzumab, and veltuzumab, and the fully human, ofatumumab. We conducted a literature search of relevant randomized clinical trials in the PubMed database and ongoing trials in Clinicaltrials.gov. Most of these trials have evaluated intravenous ocrelizumab or subcutaneous ofatumumab in rheumatoid arthritis, multiple sclerosis, or systemic lupus erythematosus. Understanding how newer anti-CD20 mAbs compare with rituximab in terms of efficacy, safety, convenience, and cost is important for guiding future management of anti-CD20 mAb therapy in autoimmune diseases.

## Introduction

Anti-CD20 monoclonal antibodies (mAbs) are used to achieve B cell depletion, and were initially developed to treat B cell proliferative disorders, including non-Hodgkin’s lymphoma (NHL) and chronic lymphocytic leukemia (CLL). Anti-CD20 mAbs have subsequently been tested and used in the treatment of the autoimmune disorder rheumatoid arthritis (RA) based on the rationale that the removal of the autoantibody producing or T cell-activating B cells would lead to clinical improvement [1]. The clear clinical benefits of anti-CD20 mAb treatment in RA, particularly in patients refractory to other available treatments, has led to the expansion of anti-CD20 mAbs for other autoimmune diseases with both T cell and B cell etiology, including systemic lupus erythematosus (SLE) and multiple sclerosis (MS).

CD20 is a transmembrane calcium channel implicated in B cell activation, proliferation, and differentiation [2]. It is present on the surface of B cells in the late pre-B cell through mature memory B cell stages. Therefore, anti-CD20 mAbs target B cells in this intermediate stage of development, sparing early pre-B cells and plasma cells, thus allowing for retention of long-term immune memory and B cell reconstitution following depletion. Due to the maintenance of antibody production by plasma cells, administration of anti-CD20 mAbs almost completely depletes peripheral B cells, but antibody levels are not dramatically reduced [3]. This suggests that the clinical benefits of this type of B cell depletion therapy may stem from the loss of other prominent B cells functions such as antigen presentation, production of inflammatory cytokines, activation of T cells, and creation of ectopic lymphoid follicles [4].

Rituximab, a murine–human chimeric anti-CD20 mAb, was the first anti-CD20 mAb developed as a therapeutic agent. It was introduced in 1997 and has since gained FDA approval for the treatment of NHL, CLL, RA, granulomatosis with polyangiitis (GPA), and microscopic polyangiitis. It was also found in some studies to be effective in other autoimmune conditions including SLE, relapsing-remitting MS (RRMS), and pemphigus [5–7]. Unfortunately, human anti-chimeric antibodies (HACA) against rituximab often develop with treatment due to rituximab’s chimeric makeup. Clinically, immunogenicity of rituximab is suspected to play a detrimental role in efficacy and tolerability.

New generations of anti-CD20 mAbs that are either humanized or fully human have been developed in recent years to address the issue of immunogenicity. However, their use could lead patients to develop human anti-human antibodies (HAHA) instead. The humanized versions include ocrelizumab, veltuzumab, and obinutuzumab, while ofatumumab is currently the only available fully human anti-CD20 mAb (see Table 1). To boost efficacy, many of the second-generation anti-CD20 mAbs have increased binding affinity to the Fc receptor on B cells and increased complement-dependent cytotoxicity (CDC) and/or antibody-dependent cellular cytotoxicity (ADCC) [8]. Their expected superiority over rituximab was one of the driving forces for their development in recent years, but another contributing factor was likely rituximab’s patent expiration in September 2016 in the United States.

**Table 1:** Characteristics of anti-CD20 mAbs

Generation	Format	Drug	Special features	FDA-approved indications
1st	Murine–human chimeric	Rituximab	Immunogenicity occurs due to chimeric nature	NHL, CLL, RA, GPA, MPA
2nd	Humanized	Ocrelizumab	Binds an overlapping epitope region as rituximab. Increased binding affinity. Enhanced ADCC and less CDC compared to rituximab [8, 32]	RRMS and PPMS
		Veltuzumab	Complementarity-determining regions are similar to rituximab. Greater binding avidity and effect on CDC than rituximab [8]	Orphan status designation for ITP and pemphigus
		Obinutuzumab	Binds to an epitope on CD20 that partially overlaps with that of rituximab. Greater ADCC than rituximab. Unlike rituximab, obinutuzumab does not stabilize CD20 in lipid rafts and thus has less CDC. More effective at direct B cell apoptosis [63]	With chlorambucil for previously untreated CLL
	Fully human	Ofatumumab	Binds to an epitope distinct from that recognized by rituximab, ocrelizumab, veltuzumab, and obinutuzumab. Greater CDC and apoptosis than rituximab [17]	Refractory or conventional therapy-intolerant CLL

Numerous clinical trials of these agents in various autoimmune conditions have been completed, and more are underway. Ongoing studies of the long-term safety profile will be needed to determine the risk of infections, malignancy, and other serious adverse effects. Obinutuzumab and ofatumumab have been FDA approved for CLL, but their use in autoimmune disorders is still being investigated. Ocrelizumab has recently been approved for RRMS, and represents the first approved treatment for primary progressive MS (PPMS). Therefore, it appears that the new generation of anti-CD20 mAbs will have important roles in the future of treatment for MS, and likely other autoimmune diseases as well. To provide a better understanding of the latest advances in the use of next-generation anti-CD20 mAbs for autoimmune diseases, including potential advantages and drawbacks, we have conducted a review of both completed and ongoing clinical trials for RA, MS, SLE, and pemphigus (see Table 2).

**Table 2:** Randomized controlled trials of next-generation anti-CD20 mAbs in autoimmune diseases

Drug	Disease	Criteria	Study	Format	Status
Ocrelizumab	RA	Patients with inadequate response to MTX	Phase I/II [15] NCT02720120	IV	Terminated
	RA	Japanese patients with inadequate response to MTX	Phase I/II [15] NCT00779220	IV	Terminated early
	RA	Patients with inadequate response to etanercept and adalimumab	Phase II [15] NCT00808210	IV	Terminated
	RA	Patients on MTX	ACTION, Phase I/II [13] NCT00077870	IV	Completed
	RA	Patients on MTX	STAGE, Phase III [9] NCT00406419	IV	Terminated
	RA	Patients on MTX	FILM, Phase III [10] NCT00485589	IV	Terminated
	RA	Patients with inadequate response to TNF antagonist	SCRIPT, Phase III [12] NCT00476996	IV	Terminated
	RA	Patients with inadequate response to MTX	FEATURE, Phase IIb [11] NCT00673920	IV	Terminated
	SLE	Patients with active SLE, but not LN	BEGIN, Phase IIINCT00539838	IV	Terminated early
	SLE	Patients with active LN	BELONG, Phase III [32] NCT00626197	IV	Terminated early
	RRMS	≥ 2 relapses in the last 3 years with ≥ 1 in the past year and ≥ 6 T2 lesions per MRI, or 2 relapses in the last year	Phase II [24] NCT00676715	IV	Completed
		≥ 2 relapses in the last 2 years, 1 relapse in the last year, or MRI consistent with MS	OPERA I and II, Phase III [22] NCT01247324, NCT01412333	IV	Approved
	PPMS	Patients meeting revised McDonald criteria and disease duration < 15 years if EDSS > 5.0, < 10 years if EDSS ≥ 5.0	ORATORIO, Phase III [23] NCT01194570	IV	Approved
	RRMS	Patients meeting revised McDonald criteria, ≥ 1 release in last year, disease duration ≤ 3 years, EDSS ≤ 3.5	Phase III NCT03085810	IV	Recruiting
	RRMS PPMS	Patients from OPERA I, OPERA II, or ORATORIO; clinically stable	Phase III NCT01765361	IV	Completed
	RRMS	Patients meeting revised McDonald criteria, EDSS ≤ 5.5, ≥ 1 immunization against TT, DT, or DTaP	Phase IIIB NCT02545868	IV	Ongoing
	RRMS	Patients meeting revised McDonald criteria, disease duration ≤ 12 years, suboptimal response to other DMT	Phase III NCT02637856	IV	Recruiting
	RRMS	Patients meeting revised McDonald criteria, disease duration ≤ 12 years, suboptimal response to other DMT	Phase III NCT02637856	IV	Recruiting
Ofatumumab	RA	Patients with inadequate response to DMARDs	Phase I/II [18] NCT00291928	IV	Completed
	RA	Biologically naïve patients who failed at least one DMARD	OFA111752, Phase II extension [17] NCT00655824	IV	Terminated
	RA	Biologically naïve patients who failed MTX	OFA110635, Phase III [16] NCT00611455	IV	Terminated
	RA	Biologically naïve patients who failed TNF antagonist	OFA110634, Phase III [17] NCT00603525	IV	Terminated
	RA	Patients who failed MTX	Phase I/II [19] NCT00686868	SC	Completed
	RRMS	≥ 2 relapses in the last 2 years, or ≥ 1 relapse in the last 1–2 years and ≥ 1 T1 GEL in the last year	Phase II [25] NCT00640328	IV	Completed
		≥ 1 relapse in the last year, ≥ 2 relapses in the last 2 years, or ≥ 1 relapse in the last 2 years with GEL in the last year	MIRROR, Phase II [26] NCT01457924	SC	Completed
		Patients with at least 1 relapse in the last year, 2 relapses in 2 years, or GEL MRI in last year	ASCLEPIOS I and II, Phase III NCT02792218, NCT02792231	SC	Recruiting
	Pemphigus vulgaris	Patients with moderate or severe disease who failed a steroid taper	Phase III NCT01920477, NCT02613910	SC	Terminated
Obinutuzumab	SLE	Patients with active LN	Phase II NCT02550652	IV	Recruiting
Veltuzumab	RA	Patients who failed MTX alone or MTX plus TNF antagonist	VELVET, Phase II NCT01390545	SC	Terminated redesigned

## Anti-CD20 mAbs in rheumatoid arthritis

RA is a common autoimmune, inflammatory disease of the joints that affects up to 1% of the US population. Rituximab is FDA approved for use with methotrexate (MTX) in RA patients who have had inadequate results with a tumor necrosis factor (TNF) antagonist. The development of next-generation anti-CD20 mAbs for use in autoimmune disease had initially focused on RA due to its relatively high prevalence.

### Ocrelizumab in RA

The use of intravenous (IV) ocrelizumab for the treatment of RA was studied in four phase III, international, randomized, double-blind, placebo-controlled trials: SCRIPT, STAGE, FILM, and FEATURE. STAGE [9], FILM [10], and FEATURE [11] studied ocrelizumab in RA patients on stable MTX who had inadequate responses to MTX, while SCRIPT [12] studied ocrelizumab in RA patients taking MTX or leflunomide who had inadequate responses to at least one TNF antagonist. Patients in the STAGE, FILM, and SCRIPT studies continued taking their stable doses of MTX or leflunomide and were assigned to receive ocrelizumab at 200 mg × 2, 500 mg × 2, or placebo × 2 at baseline and at week 24. FEATURE had a different study design, with patients randomized to receive ocrelizumab at 200 mg × 2, 500 mg × 1 (single infusion), or placebo × 2. At 24 weeks, dual infusion groups were re-randomized to either ocrelizumab dosing regimen. At 48 weeks, the open-label period began and patients received ocrelizumab at 400 mg × 1.

All phase III trials met their primary endpoints for all doses of ocrelizumab, with the exceptions of the FEATURE single infusion group and FILM, which was terminated prematurely. Compared to placebo, a greater percentage of ocrelizumab-treated patients in the FEATURE study achieved the American College of Rheumatology 20% criteria for improvement (ACR20) at week 24. Similarly, the STAGE and SCRIPT studies showed a greater percentage of ocrelizumab-treated patients with ACR20 at weeks 24 and 48, compared with placebo. However, SCRIPT also showed that joint progression on X-ray based on the modified Sharp/van der Heijde score was only slowed in the higher dose 500 mg ocrelizumab group. Finally, FILM found a reduced change from baseline in the modified Sharp/van der Heijde score at week 52, although its original primary endpoint was at 104 weeks.

SCRIPT and STAGE showed that the number of CD19^+^ peripheral B cells was higher in the ocrelizumab 200 mg group compared to the 500 mg group, at weeks 24 and 48. This was consistent with results in ACTION, an earlier phase II study of ocrelizumab in RA patients on MTX [13]. These findings suggest that the higher ocrelizumab dose is more effective in prolonged peripheral B cell depletion. In FEATURE, greater B cell repletion was found in the single infusion group at week 24, suggesting that the double infusion administration is more effective in prolonged B cell depletion.

In terms of immunogenicity, at pre-infusion baseline, 0.6% of all patients were HAHA positive, which was expected given the assay used [14]. Across all four trials, a low proportion of patients developed HAHA positivity during the double-blind placebo-controlled periods. HAHA incidence was comparable between the ocrelizumab plus MTX and placebo plus MTX groups, ranging from 1.0 to 4.7% in each treatment arm. In FEATURE, HAHA incidence ranged from 0% in placebo-treated patients who then received ocrelizumab at 200 mg × 2 to 10.7% in placebo-treated patients who then received ocrelizumab at 400 mg × 1. There was no association between HAHA positivity and CD19^+^ B cell counts or Disease Activity Score in 28 joints. Of the three patients who had a serious infusion-related reaction (IRR), none were HAHA positive. Meanwhile, of the patients who were HAHA positive, four had mild grade 1 or 2 IRRs.

An analysis of all the phase III trials SCRIPT, STAGE, FILM, and FEATURE concluded that ocrelizumab at doses of 500 mg plus MTX treatment groups were associated with a higher rate of serious infections compared to placebo plus MTX, whereas ocrelizumab 200 mg doses plus MTX did not show higher serious infection rates [9]. During the double-blind placebo-controlled period, three deaths occurred in the ocrelizumab 500 mg dose group due to respiratory failure secondary to pneumonia, pneumonia complicated by sepsis, and acute myocardial infarction, while one patient in the placebo group died of mesenteric vasculitis. Serious adverse effects were more frequent in patients recruited in Asia, particularly Japan, who received ocrelizumab 500 mg doses plus MTX. This was true even after correcting for risk factors such as cardiac disease, use of oral corticosteroids at baseline, and history of diabetes. It is not clear why patients from Asia disproportionately accounted for the serious adverse events; however, Asian groups were made up of small numbers of patients and confidence intervals were wide and overlapped with other groups. Additionally, a separate phase I/II trial performed in Japan found increased infection and serious infection rates in ocrelizumab-treated groups and was terminated early [15]. Two other phase I and II trials were also terminated given poor benefit–risk profiles in RA treatment (NCT02720120, NCT00808210). After evaluating the efficacy and safety results from available trials, the sponsor ultimately decided that the benefit over current treatments, including rituximab, did not justify further development of ocrelizumab as a treatment for RA [12]. In May 2010, ocrelizumab development in RA was terminated [14].

### Ofatumumab in RA

Between January 2008 and July 2013, three clinical trials were conducted to evaluate the long-term safety and efficacy of repeated treatment cycles of IV ofatumumab in different populations of RA patients. OFA110635 was a study of IV ofatumumab in biologically naive RA patients who had failed MTX [16], while OFA110634 was a study of IV ofatumumab in biologically naive patients who had failed TNF antagonists [17]. These were both phase III trials with 24-week randomized, double-blind, placebo-controlled periods followed by 120-week open-label retreatment periods, and then safety follow-up for up to 2 years. Patients across both trials received a course of two infusions of 700 mg ofatumumab or placebo given 2 weeks apart. Patients could be re-treated if clinically indicated, and remained on their stable doses of MTX. In OFA110635, a greater proportion of ofatumumab-treated patients, 50% vs. 27% with placebo at 24 weeks (*p* < 0.001), achieved ACR20 responses. OFA110634 had similar results at 24 weeks, with ACR20 in 42% of patients with ofatumumab vs. 19% with placebo (*p* value was not published). OFA111752 was a phase II extension trial with a 130-week open-label period and safety follow-up [16]. The study followed RA patients who failed at least one disease-modifying antirheumatic drug (DMARD) who were enrolled in a prior phase I/II dose-ranging trial. They had originally received ofatumumab at 300, 700 or 1000 mg doses or placebo [18]. In the extension phase, patients received individualized open-label ofatumumab retreatment of 700 mg × 2 intravenous infusions given 2 weeks apart, ≥ 24 weeks after their first course and ≥ 16 weeks after further courses. The primary endpoint of the phase II extension was time to treatment withdrawal. While preliminary results were promising, the trials were discontinued prematurely due to the sponsors deciding to shift ofatumumab development to a subcutaneous (SC) formulation.

These studies showed that IV ofatumumab was effective in decreasing disease activity, and inducing remission in RA patients who had inadequate responses to MTX, TNF antagonists, or other DMARDs [17]. Additionally, individualized retreatment with ofatumumab infusions at a dose of 700 mg × 2 was efficacious and safe in RA patients. Given premature termination, only limited data on anti-drug antibodies are available. All 92 samples from patients tested in OFA111752 were negative for anti-drug antibodies, and there were no major observed differences across trials involving RA patients who were biologically naive, DMARD refractory, or those who had prior exposure to TNF antagonists. Across trials, mild to moderate IRRs were common (48–79%), while serious IRRs were uncommon (< 1% to 5% of patients). Given frequent IRRs, demonstrated techniques to reduce the IRRs were implemented, including increased infusion volume, increased infusion time, and premedication with IV glucocorticoids. Two deaths occurred, one due to fulminant hepatitis B virus infection and the other due to interstitial lung disease.

As an alternative to the IV route and to eliminate the need for premedication with glucocorticoids, a SC formulation was investigated with the rationale that SC ofatumumab could achieve a slower rate of absorption and B cell depletion and potentially improve safety and tolerability. The sponsors began testing a SC formulation of ofatumumab in a 24-week phase I/II, single-blind trial with extended follow-up for up to 2 years to evaluate its safety and tolerability in RA patients who failed MTX [19]. Patients received a single SC injection of ofatumumab at 0.3, 3, 30, 60, or 100 mg doses or placebo with oral antihistamine and acetaminophen premedication. Full target B cell depletion was achieved in patients who received a single dose of 30, 60, or 100 mg. Doses up to 60 mg were tolerated without glucocorticoid premedication. Injection-related systemic adverse events, including nausea, fever, dizziness, and headache, were mostly mild or moderate and more common in the ofatumumab-treated group, 48% vs. 25% with placebo. Notably, adverse events occurred in all three patients receiving the 100 mg dose of ofatumumab. Upper respiratory infection was a common adverse event, but no serious infections occurred. The doses used in this study are remarkably lower than those used in IV formulations, and still effective at depleting peripheral B cells. Doses between 30 to 60 mg appear to be well tolerated. The ability to use these lower doses could have major implications for the delivery, cost, and accessibility of anti-CD20 mAbs, but further studies will be needed to determine efficacy, optimal dosing schedule, and long-term safety in RA patients.

### Veltuzumab in RA

A dosing study of SC veltuzumab was initiated in patients with RA, but the sponsor decided to terminate it and redesign the protocol (NCT01390545).

## Anti-CD20 mAbs in multiple sclerosis

MS is a chronic demyelinating autoimmune disease of the central nervous system which affects approximately 2.5 million people worldwide. After success in RA trials, rituximab was investigated as a treatment for RRMS and PPMS. Results of phase II trials were favorable in RRMS [20] and a subset of PPMS patients who were less than 51 years old and/or had gadolinium enhancing lesions (GEL) [21]. However, the sponsor decided to shift the focus to developing ocrelizumab.

### Ocrelizumab in MS

Three recent landmark clinical trials of ocrelizumab in MS have been completed. OPERA I and II were identical 96-week phase III, multicenter, randomized, double-blind, double-dummy, active-controlled, parallel-group trials investigating ocrelizumab vs. interferon β-1α (IFN β-1α) in RRMS [22]. Ocrelizumab IV infusions were administered at a dose of 600 mg every 24 weeks, while IFN β-1α SC injections were administered at 44 µg three times/week. The ocrelizumab group met its primary endpoint, with statistically significant reductions in annualized relapse rate at 96 weeks. In OPERA I, ocrelizumab-treated patients had a 46% lower relapse rate with 0.16 vs. 0.29 (*p* < 0.001), and similarly in OPERA II, there was a 47% lower rate with 0.16 vs. 0.29 (*p* < 0.001). Ocrelizumab-treated patients also had a statistically significantly lower percentage of patients with disability progression at 12 and 24 weeks, lower mean number of GEL on T1-weighted MRI, and better MS functional composite scores. Immunogenicity was limited, with only 0.4% of ocrelizumab-treated patients across the two trials developing anti-drug antibodies. In contrast, neutralizing IFN β-1α antibodies were found in 21.3% of IFN β-1α-treated patients. Across the two trials, IRRs were more common in the ocrelizumab group at 34.3%, but only occurred in 9.7% of patients in the IFN β-1α group. Serious infections were comparable, occurring in 1.3% of the ocrelizumab group vs. 2.9% in the IFN β-1α group, as were neoplasms which occurred in 0.5% vs. 0.2%, respectively. Overall, ocrelizumab was found to be superior to IFN β-1α in terms of efficacy with a similar safety profile, aside from common IRRs with ocrelizumab.

ORATORIO was a 120-week phase III, randomized, parallel-group, double-blind, placebo-controlled trial that studied ocrelizumab vs. placebo in PPMS [23]. Patients were assigned to receive IV ocrelizumab at 600 mg or IV placebo every 24 weeks. ORATORIO met its primary endpoint, with a statistically significant decrease in the percentage of patients with disability progression at 12 weeks at 32.9% with ocrelizumab vs. 39.3% with placebo (*p* = 0.03). Similar trends followed at 24 and 120 weeks. By week 120, fewer ocrelizumab-treated patients had declined performance on the timed 25-foot walk, total volume of brain lesions on T2-weighted MRI decreased, and there was a lower percentage of brain-volume loss. There was, however, no significant difference in the change in the Physical Component Summary score of the 36-Item Short-Form Health Survey. Adverse events that were more common with ocrelizumab than placebo were IRRs, upper respiratory tract infections, and oral herpes infections. There was an imbalance of neoplasms, which occurred in 2.3% of ocrelizumab-treated patients vs. 0.8% with placebo, but there was no clinically significant difference in the rates of serious adverse events and serious infections. Although statistically significant improvements in clinical and MRI progression were demonstrated in ORATORIO, the effects could be considered modest. Additionally, it is important to note that the ORATORIO trial was not powered to demonstrate a difference in the subgroup analysis of efficacy between patients with and without GEL at baseline. Given that earlier trials of rituximab in PPMS suggested its efficacy was limited to a subset of patients with GEL [21], it is possible that ocrelizumab might benefit PPMS patients with an inflammatory-predominant form of the disease more than the neurodegenerative form. Further studies are required to determine which subset of PPMS patients benefit most from ocrelizumab.

While ocrelizumab in RA was met with safety concerns regarding serious infections (“Ocrelizumab in RA”), this has not been the case with MS trials to date. However, safety concerns were raised during the open-label extension phases of ocrelizumab in MS trials when an imbalance of malignancies presented. In the ocrelizumab-treated MS patients across all four studies, including the phase II study in RRMS (NCT00676715) [24], incidence of a first neoplasm was 0.40 per 100 patient-years of exposure vs. 0.20 out of 100 patient-years in the comparator groups (placebo and IFN β-1α) [23]. Ocrelizumab has recently been granted FDA approval for both RRMS and PPMS, and represents the first FDA-approved treatment for PPMS. Further studies to better characterize the utility of ocrelizumab are underway, including use in early-stage RRMS (NCT03085810), use in patients with RRMS and inadequate response to other disease-modifying therapies (DMTs) (NCT02637856), immune response to vaccines after a dose of ocrelizumab (NCT02545868), and effect on the neurodegenerative subset of RRMS and PPMS patients using subsamples from the ORATORIO and OPERA I and II trials (NCT01765361). Ocrelizumab currently carries a black box warning for hepatitis B reactivation, but continued monitoring will be necessary to appropriately assess ocrelizumab’s long-term safety profile in MS.

### Ofatumumab in MS

Ofatumumab has undergone two phase II trials in RRMS. One study was designed as a 48-week phase II, randomized, double-blind, placebo-controlled trial assessing safety and preliminary efficacy of IV ofatumumab. It compared ofatumumab infusions at 100, 300, and 700 mg doses vs. placebo for weeks 0–24, at which point patients crossed over to the alternative study arm for weeks 24–48 [25]. MRI data suggested efficacy at all doses studied, which were lower than the 1000 mg doses of rituximab used in off-label RRMS treatment, potentially due to ofatumumab’s increased potency over rituximab. IRRs were the most common adverse event and more common in the ofatumumab-treated group, occurring in 88.5% of patients with ofatumumab vs. 8.3% with placebo. No cases of opportunistic infections or neoplasms were reported. No immunogenicity was detected in any of the ofatumumab-treated patients. Overall, IV ofatumumab had a favorable safety profile and promising results.

Due to the high rate of IRRs with IV ofatumumab and the favorable safety profile demonstrated by a phase I/II trial for SC ofatumumab in RA patients [19], a study of SC ofatumumab in MS was conducted. The MIRROR trial was a 24-week phase II, randomized, double-blind, placebo-controlled, parallel-group, dose-ranging study in RRMS in which ofatumumab was administered as SC injections [26]. Patients received 3, 30, or 60 mg SC doses every 12 weeks, 60 mg SC every 4 weeks, or placebo. At week 12, patients on placebo received a single dose of 3 mg SC ofatumumab. Data from weeks 0–12 showed a statistically significant reduction in new T1 GEL of 65% (*p* < 0.001) compared to placebo. Injection-related reactions were the most common adverse event, occurring in 52% of ofatumumab-treated patients vs. 15% of placebo. Five serious adverse events were reported during treatment, but there were no cases of opportunistic infections.

The sponsor is currently recruiting for phase III trials, ASCLEPIOS I and II, of SC ofatumumab vs. oral teriflunomide in relapsing MS [27]. SC injection is a new route of administration of anti-CD20 mAbs that surely has the potential to compete with other injectable MS treatments, such as interferon beta’s, and carries an advantage over-infused anti-CD20 mAbs.

## Anti-CD20 mAbs in systemic lupus erythematosus

SLE is a chronic inflammatory disease with prominent immunologic abnormalities including antinuclear antibodies that can impact virtually every organ. When SLE affects the kidneys, it is referred to as lupus nephritis (LN). Currently, in SLE immunotherapy, there is no anti-CD20 mAb demonstrated to be effective in randomized controlled trials [5]. Rituximab has been effective in several open-label, uncontrolled studies, and a systematic review found improved outcomes with rituximab [28]. However, neither of the randomized controlled trials, EXPLORER [29] and LUNAR [30], met primary endpoints. The EXPLORER trial, which studied rituximab in patients with active extra-renal SLE, involved excessive use of background immunosuppressants which may have been limiting. LUNAR studied rituximab in patients with active proliferative nephritis, and all patients received concomitant mycophenolate mofetil (MMF) and corticosteroids [30]. While the use of rituximab in SLE is controversial, it is nevertheless recommended in the American College of Rheumatology guidelines as a consideration for treatment of refractory cases of SLE [31].

### Ocrelizumab in SLE

Two phase III studies of ocrelizumab in patients with SLE were started, and then terminated prematurely. BEGIN was a phase III study of ocrelizumab in patients with active, non-renal SLE, but was terminated early when its sponsor decided ocrelizumab was unlikely to benefit this population (NCT00539838). Ocrelizumab was then investigated in BELONG, a multicenter, randomized, double-blind, placebo-controlled, parallel-group phase II study, in SLE patients with progressive LN [32]. Patients were randomized to receive 400 or 1000 mg ocrelizumab or placebo: at baseline, after 2 weeks, then every 4 months thereafter. All patients additionally received either cyclophosphamide or MMF. The cyclophosphamide regimen was based on the EUROLUPUS trial, which studied low-dose cyclophosphamide in SLE treatment [33]. The BELONG study was designed to continue for at least 2 years, with the primary endpoint as renal response at 48 weeks. The trial was terminated early due to an imbalance in rate of serious infections in the ocrelizumab group receiving MMF. 381 patients had been recruited, and 221 patients had passed the 32-week point and were assessed. The renal response rate was 63% and 51% in the combined ocrelizumab and placebo groups, respectively. Upon subgroup analysis, there was a greater treatment effect with adding ocrelizumab to the EUROLUPLUS cyclophosphamide regimen, with renal response of 65.7% compared to 42.9% with cyclophosphamide alone [33, 34]. The difference was much less pronounced in the MMF regimen, with renal response rates of 67.9% with ocrelizumab vs. 61.7% with MMF alone. This can be explained by the higher response rate with MMF in general and could shed light on why the LUNAR study of rituximab in SLE patients with proliferative lupus nephritis, which also used MMF, did not reveal clinical benefits.

### Obinutuzumab in SLE

A phase II trial of the anti-CD20 mAb obinutuzumab plus MMF vs. placebo plus MMF in SLE patients with LN is actively recruiting patients (NCT02550652). Since the benefits of rituximab or ocrelizumab have not been prominent with concurrent MMF treatment, it will be interesting to see if this will also be the case in this trial, which has similar treatment group regimens.

### Veltuzumab in SLE

Veltuzumab was effective in a 2005 case study of a patient with severe, resistant SLE who failed conventional therapies and initially responded to rituximab. The patient then had clinical worsening of her disease and developed high levels of anti-rituximab antibodies. She received veltuzumab on a compassionate-use basis and responded well, with B cell depletion and remarkable clinical improvement [35]. It is unclear whether this is an isolated case or could be generalized to other patients with SLE refractory to rituximab, since veltuzumab has not been in development for SLE.

## Anti-CD20 mAbs in pemphigus

Pemphigus vulgaris and pemphigus foliaceus are chronic autoimmune blistering diseases that can result in significant morbidity and death. After initial reports of the benefits of rituximab in paraneoplastic pemphigus in patients treated with rituximab for B cell lymphoma, rituximab was found to be effective for pemphigus vulgaris and pemphigus foliaceus in retrospective studies, case series, and small uncontrolled trials [36–39]. The optimal dosing of rituximab for pemphigus has not yet been determined. Rituximab is used in refractory cases of pemphigus and is usually prescribed concomitantly with other systemic immunosuppressive therapies.

### Veltuzumab in pemphigus

SC veltuzumab was used on a compassionate-use basis in a patient with refractory pemphigus vulgaris. Two SC injections of 325 mg veltuzumab were given 2 weeks apart, which led to a complete remission for 2 years. At 2 years, the patient relapsed and received a second cycle of veltuzumab at the same dosing regimen. Remission was maintained 9 months later at the end of the follow-up period [40]. Veltuzumab was granted Orphan Status Designation by the FDA for pemphigus in 2015.

### Ofatumumab in pemphigus

There was a phase III randomized clinical trial of SC ofatumumab in pemphigus that recruited 35 patients, but it was terminated after ofatumumab was acquired by a different sponsor (NCT01920477).

## Efficacy of anti-CD20 mAbs

Next-generation anti-CD20 mAbs have been shown to be more potent than rituximab in vitro; however, this has not been confirmed in vivo. Thus far, there have been no head-to-head trials of rituximab against the newer agents. However, a new phase III trial is now underway to directly compare safety profile and tolerability in patients with RRMS on IV rituximab who switch to IV ocrelizumab vs. those who continue on rituximab (NCT02980042). More of these types of direct comparison studies will help determine whether there are true advantages, in terms of safety and efficacy, to the new anti-CD20 mAbs.

The development of antibodies to chimeric rituximab has raised concerns that immunogenicity of anti-CD20 mAbs could impact efficacy, tolerability and risk of IRRs. A safety analysis of RA patients treated with rituximab in combination with MTX in clinical trials showed that 11% of patients developed a positive titer for HACA’s at least once during treatment with rituximab [41]. However, the presence of HACA was not associated with IRRs or reduced efficacy upon retreatment. Additionally, in the HERMES trial, a phase II study of rituximab in RRMS, HACAs were detected in 24.6% of rituximab-treated patients at week 48, but there was no association between presence of HACA and efficacy or adverse effects [7]. On the other hand, there is evidence that suggests immunogenicity can affect the efficacy of rituximab in some patients. One case study described a young female patient with severe resistant SLE who initially responded to rituximab, but eventually, rituximab treatment became inadequate to control her disease, and high levels of anti-rituximab antibodies were found [35]. She then received veltuzumab on a compassionate-use basis and achieved disease remission. Therefore, it is possible that under dire situations when tolerance to an anti-CD20 mAb has developed, switching to another anti-CD20 mAb could potentially be useful.

Although there are no direct comparisons in immunogenicity between rituximab and the newer anti-CD20 mAbs, rates of immunogenicity appear lower with the newer anti-CD20 mAbs. In the 48-week phase II study of ofatumumab in RRMS, none of the patients tested HAHA positive [25], while in the phase II study of ocrelizumab in RRMS the incidence of HAHA was similar between the ocrelizumab and placebo groups (2–3%) [24]. Furthermore, even in cases where HAHA develop there has not been a consistent association between immunogenicity and lack of efficacy or adverse effects. For example, in a phase I study of veltuzumab in idiopathic thrombocytopenic purpura (ITP), many patients developed immunogenicity to veltuzumab, but still showed clinical improvement [40].

Additionally, there is some evidence that the extent of autoimmune disease progression at time of first treatment can affect the efficacy of anti-CD20 mAbs. Studies suggest a possible enduring advantage of early control of pathogenic B cells in autoimmune disease progression, particularly in myasthenia gravis (MG) and ITP. A meta-analysis of uncontrolled observational studies of rituximab in patients with MG showed a trend toward an inverse correlation between the duration of disease and rate of response [42]. Similarly, in the phase I study of veltuzumab in ITP patients, there was a trend toward a longer median time to relapse in patients who had ITP for a year or less [43]. If similar results are found in the context of other diseases then it could alter future treatment management decisions, as physicians typically start newly diagnosed patients on the least potent and also safest treatment options first. If early treatment with anti-CD20 mAbs can curtail disease progression and demonstrate a good safety profile, anti-CD20 mAbs may eventually become a first-line therapy in autoimmune diseases.

## Safety profile of anti-CD20 mAbs

If anti-CD20 mAbs are to become the new standard of care for a given autoimmune disease, they will need to offer an excellent safety profile. IRRs are the most common adverse events of anti-CD20 mAb infusions, including rituximab and newer anti-CD20 mAbs. IRRs are partially attributed to cytokine release after pronounced B cell depletion upon the first infusion [44]. IRRs occur primarily after the first infusion, are most often mild or moderate in severity, and rarely require discontinuation of treatment. Patients may experience local and systemic reactions including urticaria, angioedema, headache, nausea, fever, chills, rigors, and even bronchospasm and hypoxia in rare severe cases. Humanized and human anti-CD20 mAbs are theorized to cause reduced incidence and lower severity of IRRs compared to rituximab due to decreased immunogenicity. However, this has not been directly compared, and IRRs have continued to be the most common adverse event with infusions of newer anti-CD20 mAbs.

SC formulations of anti-CD20 mAbs appear to be the next step in development, which could reduce IRRs, eliminate the need for premedication with glucocorticoids, and offer a potentially more convenient administration method. A SC formulation of rituximab has already been developed and approved in Europe in 2014 for use in patients with follicular lymphoma and NHL, and in 2016 for CLL. Ofatumumab is also being developed as a SC formulation for use in RA and RRMS, and a SC formulation of veltuzumab was in development for RA patients with plans to start a new trial after the protocol is redesigned [19, 26]. SC rituximab is administered over 5 min, and the patient needs to be monitored for IRRs for at least 15 min after the injection. This is significantly less time compared with IV rituximab, with which patients need to be monitored throughout the infusion process lasting up to 6 h [45]. The SC route is preferred by 93% of CLL patients and 95% of nurses, according to part one of the phase 1b SAWYER study investigating SC versus IV rituximab in patients with previously untreated CLL [46]. Adverse events are more frequent with the SC route compared with IV, but the most common adverse events are low-grade, local injection-site reactions including erythema and pruritus, which typically resolve within 1 to 2 days [45]. In November 2016, the FDA accepted the drug maker’s biologics license application for a SC formulation of rituximab for several hematologic malignancy indications for use in the US.

The long-term consequences of B cell depletion are not known, but agents that interfere with the immune system raise concerns for infection and malignancy. Progressive multifocal leukoencephalopathy (PML) is a serious opportunistic infection that is of great concern with anti-CD20 mAb use. It is a rare and often debilitating and fatal demyelinating disease of the central nervous system caused by JC virus infection of oligodendrocytes, and is typically only seen in patients with severely compromised immune systems. The broader use of immunosuppressive and biologic agents, including agents used in autoimmune disease treatment, has presented the issue of iatrogenic PML [47, 48]. Cases of PML have been reported with rituximab use in patients with lymphoma, RA, SLE, GPA, and MG [49, 50]. Rituximab does carry black box warnings for hepatitis B reactivation and PML. However, there are differences in the incidences of PML in different subsets of patients using anti-CD20 mAb treatment. The incidence of PML in rituximab-treated RA patients is 1:30,000 [51]. Notably, PML cases in this subgroup occurred in patients who were previously or concurrently treated with additional immunosuppressive therapy, such as MTX. Immunosuppressed SLE patients have been found to be at a higher risk of developing PML than patients with other rheumatic disorders, with rates of PML in SLE patients at 4 per 100,000 admissions compared to 0.4 for RA patients and 2 for other connective tissue disorders [52]. Several of the reported cases of PML in SLE patients have been attributed to rituximab [53]. In contrast, no cases of PML have been reported in MS patients, including those with RRMS, SPMS, and PPMS, who have received rituximab in clinical trials, or with off-label use [7, 21, 54]. Since immunosuppression increases the risk of PML, the common use of additional immunosuppressants in RA and SLE, but not MS, could help explain this disparity in risk. Furthermore, anti-CD20 mAbs actually carry a lower risk for PML than some other MS treatments. For example, the incidence of PML using natalizumab treatment in MS and Crohn’s disease ranges from 1:100 to 1:1000 [51]. At this point, it is expected that the newer anti-CD20 mAbs will carry similar PML risk profiles as rituximab.

Ofatumumab was FDA approved for use in CLL in 2014, and carries black box warnings for reactivation of hepatitis B and PML. A PML-related death has occurred in an ofatumumab-treated patient with history of CLL and other comorbid malignancies [55]. Obinutuzumab was FDA approved in 2013 for CLL and also carries black box warnings for hepatitis B reactivation and PML [56]. Veltuzumab is being developed for hematologic malignancies and has orphan status approval for ITP. No PML cases have been reported, but it is too early to make an assessment.

While the infection and PML risk is higher for anti-CD20 mAb-treated patients with RA and SLE, the risk of developing malignancies appears to be higher for MS patients. An analysis of the phase II and III randomized clinical trials of ocrelizumab in RRMS and PPMS revealed an imbalance of malignancies in the ocrelizumab-treated groups [22, 23]. Meanwhile, an analysis of rituximab trials in RA patients found no increased risk of malignancy compared to other RA patients or the general US population [41]. Malignancy is a concern for patients with autoimmune diseases who would likely need repeated treatments over their chronic disease course. Now that ocrelizumab has been FDA approved for RRMS and PPMS, continued monitoring will be necessary to determine whether the risk of malignancy will warrant future warnings or limitations of use.

## Prospective cost of anti-CD20 mAbs

The effect on price competition of introducing new anti-CD20 mAbs into the market, particularly those using SC administration at lower doses and biosimilars, is an open question. Historically, in the context of MS, the introduction of new disease-modifying agents has paradoxically raised prices of older drugs. Rituximab’s patent expired in Europe in February 2013 and more recently in the US in September 2016. Meanwhile, a biosimilar of rituximab, Acellbia, has already undergone phase III trials and was approved by the Ministry of Health of the Russian Federation in May 2014 [57]. The FDA has approved four biosimilars (Zarxio, Inflectra, Erelzi, and Amjevita) since 2015, and biosimilars of rituximab are in the pipeline [58]. Rituximab’s patent expiration also parallels the pharmaceutical industry’s enthusiasm in recent years to develop the newer generation anti-CD20 mAbs. In 2015, rituximab costs about $30,000 annually for the treatment of RA using the dosing schedule of two infusions of 1000-mg doses totally given 2 weeks apart every 6 months [59]. Off-label use for MS has used a similar dosing schedule, so a similar price would be expected. This is considerably less than other disease-modifying MS drugs which are currently estimated to cost approximately $70,000 to $80,000 or more annually [60]. This includes the first-generation treatments that were approved two decades ago: IFN β-1β, IFN β-1α, and glatiramer acetate, which have actually seen the most dramatic annual price increases, ranging from 21.0% to 35.7% [61]. Notably, the drug maker of the newly approved ocrelizumab has set the annual cost of twice-a-year infusions to the price of $65,000 [62]. Although the stated price is within the range or less than other current MS drugs on the market, it is still significantly more expensive than rituximab. If biosimilars of rituximab become FDA approved in the future, this may drive price competition and lower the cost of rituximab. Even so, it is unclear if rituximab or its biosimilars would be a viable option for MS patients since the FDA has now approved ocrelizumab for RRMS and PPMS. Ofatumumab has not yet been approved for treatment in autoimmune diseases, but with ongoing trials of SC ofatumumab in MS and RA, it will likely become a major player if approved. The SC administration is likely to be a huge selling point for both physicians and patients, so it will be interesting to see if that is reflected in a higher cost.

## Conclusion

Anti-CD20 mAbs have demonstrated that B cells are important players in autoimmune diseases beyond their role in the generation of autoantibodies. A better understanding of how B cells participate in the development of a particular autoimmune condition will eventually allow for the development of more targeted B cell-mediated therapies. In the meantime, the latest generation of anti-CD20 mAbs is demonstrating effectiveness for some autoimmune diseases such as RA and RRMS, in which the first generation was also beneficial, but appears to offer a better safety profile in terms of immunogenicity. Furthermore, the latest formulations of ofatumumab offer the more convenient and better tolerated method of SC administration, while ocrelizumab offers the first FDA-approved treatment for PPMS. Ultimately, factors including efficacy, safety, tolerability, administration method, accessibility, cost, and patient preference will need to be considered on an individual basis to determine the best approach to the use of anti-CD20 mAbs in autoimmune disease treatment.
